# Practical considerations when providing palliative care to patients with neuroendocrine tumors in the context of routine disease management or hospice care

**DOI:** 10.1530/ERC-22-0226

**Published:** 2023-06-21

**Authors:** Jaydira Del Rivero, Josh Mailman, Michael W Rabow, Jennifer A Chan, Sarah Creed, Hagen F Kennecke, Janice Pasieka, Jennifer Zuar, Simron Singh, Lauren Fishbein

**Affiliations:** 1 Developmental Therapeutics Branch, National Cancer Institute, National Institutes of Health, Bethesda, USA; 2 NorCal CarciNET Community, Oakland, California, USA; 3 Department of Internal Medicine, Division of Palliative Medicine, University of California, San Francisco, San Francisco, California, USA; 4 Harvard Medical School, Program in Carcinoid and Neuroendocrine Tumors, Department of Medical Oncology, Dana-Farber Cancer Institute, Boston, Massachusetts, USA; 5 Good Shepherd Community Care, Harvard Kennedy School, Natick, Massachusetts, USA; 6 Providence Cancer Institute Franz Clinic, Portland Providence Medical Center, Portland, Oregon, USA; 7 Department of Surgery, Section of General Surgery, University of Calgary, Cumming School of Medicine, Calgary, Canada; 8 Department of Internal Medicine, Division of Geriatrics and Palliative Medicine, Alpert Medical School, Providence, Rhode Island, USA; 9 Odette Cancer Centre, Sunnybrook Health Sciences Centre, Toronto, Ontario, Canada; 10 Department of Medicine, Division of Endocrinology, Metabolism and Diabetes, Department of Biomedical Informatics, University of Colorado School of Medicine, Aurora, Colorado, USA

**Keywords:** neuroendocrine tumors, palliative care, hospice care, end of life, management

## Abstract

This serves as a white paper by the North American Neuroendocrine Tumor Society (NANETS) on the practical considerations when providing palliative care to patients with neuroendocrine tumors in the context of routine disease management or hospice care. The authors involved in the development of this manuscript represent a multidisciplinary team of patient advocacy, palliative care, and hospice care practitioners, endocrinologist, and oncologists who performed a literature review and provided expert opinion on a series of questions often asked by our patients and patient caregivers affected by this disease. We hope this document serves as a starting point for oncologists, palliative care teams, hospice medical teams, insurers, drug manufacturers, caregivers, and patients to have a frank, well-informed discussion of what a patient needs to maximize the quality of life during a routine, disease-directed care as well as at the end-of-life.

## How do we define palliative care, and when should it be started?

Palliative care is a medical subspecialty that focuses on alleviating the symptoms and stress associated with serious medical illness. Palliative care is appropriate for any patient with a high symptom burden at any pointin the illness trajectory (Temel *et al.* 2010). It is highly individualized to the needs of the patient and family and should be provided concurrently with disease-directed therapy. Palliative care is a larger approach to care and should be offered to those at any point in their cancer journey. Hospice differs from palliative care since it provides care specifically at the end of life and in a place which a patient calls home, be it a private residence or institution. Palliative care seeks to treat the whole patient by managing their physical, emotional, and psychosocial distress. All practitioners should strive to provide this type of care; however, patients with complex symptoms may benefit from referral to specialist palliative care providers (Temel *et al.* 2010, Zimmermann *et al.* 2014). This can be provided in the inpatient or outpatient setting (including at home and in the community) and involves an interdisciplinary team which may include physicians, nurses, social workers, and spiritual care providers (Ferrell *et al.* 2017).

Multiple trials have shown the benefit of early palliative care referrals in oncology patients; however, there are no established guidelines on when to refer patients (Temel *et al.* 2010). Many providers as well as the American Society of Clinical Oncology consider referral within 8 weeks of diagnosis of advanced cancer to be the ideal, based on previous randomized controlled trials in patients with lung cancer (Temel *et al.* 2010, Ferrell *et al.* 2017). Patients with neuroendocrine tumors (NETs) represent a unique population, as many have metastatic disease at presentation, yet can have prolonged survival rates for years. Since these patients can have a heavy symptom burden, a common criterion for referral to palliative care is to help manage debilitating physical symptoms such as pain, dyspnea, nausea and vomiting, diarrhea, and fatigue (Hui *et al.* 2016). Patients who have progressed through multiple lines of treatment may be referred for discussions to enhance their understanding of the prognosis. Palliative care referral may also be appropriate in helping patients with advanced care planning and to elicit end-of-life preferences care to ensure that all treatments are consistent with a patient's individual goals of care (Hui *et al.* 2016, Bakitas *et al.* 2015).

## How do we define hospice, and when should it be started?

For this paper, we will use the American Medicare definition of hospice. According to the Centers for Medicare and Medicaid Services (CMS), hospice care is defined as care that is provided when a patient’s hospice doctor and primary doctor (if the patient has one) both certify that the patient has a terminal diagnosis with a prognosis of 6 months or less to live if the illness progresses in its normal course. Once a patient has elected the Medicare hospice benefit, hospice should cover all care related to the patient's terminal illness in the United States. This includes, but is not limited to, care by hospice-trained clinicians such as nurses, doctors, medical social workers, prescription drugs related to the hospice diagnosis, and medical equipment and supplies. The North America Neuroendocrine Tumor Society (NANETS), as a North American organization, recognizes that hospice care in Canada and Mexico (and in other countries in the world) may be defined differently. Additionally, some private insurances have alternative hospice models, which may allow for carve-outs of specific treatments or early election of the hospice benefit. Despite this, according to the National Hospice and Palliative Care Organization (NHPCO), most hospice patients in the United States are Medicare beneficiaries.

Studies have shown that early commencement of hospice leads to better symptom control and improved outcomes for patients and their caregivers (Cheraghlou *et al.* 2017). In many cases, patients live longer when on hospice than if continuing with traditional treatment (Connor 2007, Connor *et al.* 2007). For the patient’s caregivers, hospice provides support and resources from an interdisciplinary team including respite services when needed. Earlier hospice allows for relationship building between the hospice team, patient, and caregivers, enabling the hospice services to deliver continuous support both during the end-of-life event and for 13 months after the patient's death in the form of bereavement counseling. The biggest complaint that families shared in the Consumer Assessment of Healthcare Providers and Systems Survey is that they wished that hospice had started sooner rather than later in their loved one’s disease process, according to CMS. Seeing as Medicare states that a beneficiary can receive hospice services when two doctors certify that the patient has 6 months or less to live if the patient’s disease follows its normal course, it is the patient’s right to receive this benefit as close to 6 months prior to death as possible.

Hospice is limited to palliative treatments. For patients with NET at the end of life, starting hospice traditionally has meant forgoing treatments directed at the disease. However, NET treatments such as somatostatin analogs (SSAs) may be the most effective palliative treatments available and efforts are being made to support hospices to cover the cost and assist with the administration of such palliative treatments.

## Palliative care for patients living with neuroendocrine tumors

In general, palliative treatments from simple medications to complex interventional radiology procedures are used similarly in patients with NETs as they are for patients with non-NET cancers. In patients with NET receiving palliative care, commonly used medications for symptomatic treatment, integrative treatments, procedures for stenting and drainage, and NET-directed treatments (treating symptoms at the source) are listed in Table 1.

**Table 1:** Selected commonly used medications, integrative treatments, procedures, and NET-directed treatments in people with symptom-related NET.

Treatment type (✓ if relatively specific to palliation of NET)	Intervention	Typical indication in people with NET
Symptom management with medications	Opioids	Pain
	Co-analgesics (e.g. gabapentin, pregabalin, SNRIs, TCAs)	Pain, neuropathic pain
	Stimulants (e.g. methylphenidate, modafinil)	Fatigue
	Antidepressants (Nobels *et al.* 2016): (e.g. SNRIs (venlafaxine, duloxetine), SSRIs, mirtazapine)	Depression
	Anxiolytics (e.g. ativan)	Anxiety
✓	Carcinoid syndrome management medications: somatostatin analogs (Modlin *et al.* 2010): (e.g. octreotide sq/IM, lanreotide IM), telotristat (Pavel *et al.* 2018)	Diarrhea
✓	Ursodiol	Diarrhea from bile acid malabsorption
✓	Pancreatic enzyme replacement	Diarrhea from pancreatic insufficiency
	General anti-diarrheal (e.g. loperamide, diphenoxylate & atropine, tincture of opium)	Diarrhea
	Anti-emetics (e.g. ondansetron, prochlorperazine, olanzapine, mirtazapine)	Nausea & vomiting
	Appetite stimulants (e.g. mirtazapine)	Anorexia, weight loss
✓	Gut antibiotics (e.g. rifaximin)	GI distress from bacterial overgrowth
	Bronchodilators	Wheezing
Integrative practices	Acupuncture	Pain, nausea, fatigue
	American ginseng	Fatigue
	Medical cannabis (THC strain especially for appetite stimulation; CBD strain especially for anxiety, insomnia, and neuropathic pain)	Anorexia, nausea, anxiety, pain
	Massage	Pain
	Ice/heat	Pain
	Relaxation	Pain, anxiety
	Exercise	Fatigue, pain
✓	NET nutritional interventions (Artale *et al.* 2020): avoiding amines and serotonergic foods; supplementing for vitamin deficiencies	Carcinoid syndrome or malabsorption symptoms
Palliative procedures	Surgery	Debulk or remove sites of tumor-causing symptoms
	Paracentesis	Ascites
	Thoracentesis	Pleural effusions
	Biliary stenting	Hepatic obstruction
	Gastric decompression (e.g. with nasogastric tube)	Partial small bowel obstruction

NET-directed treatments (management of disease-causing symptoms)	Chemotherapy (e.g. capecitabine, temozolomide, 5-fluorouracil, streptozocin, doxorubicin)	Pain, diarrhea
	Targeted therapy (e.g. everolimus (mTOR inhibitor), sunitinib (tyrosine kinase inhibitor))	Pain, diarrhea
	Immunotherapy (e.g. interferon alfa-2b)	Pain, diarrhea
✓	Peptide receptor radionuclide therapy (PRRT)	Pain, diarrhea
	Radiation	Pain, diarrhea
	Surgery (Goretzki *et al.* 2018, Hallet *et al.* 2021)	Pain, diarrhea
✓	Radiofrequency ablation (RFA)	Pain
✓	Hepatic artery embolization	Pain

SNRI, selective serotonin norepinephrine reuptake inhibitors; SSRI, selective serotonin reuptake inhibitors; TCA, tricyclic antidepressants.

The indication for palliative interventions in NET patients is to improve the quality of life without respect for other goals of care, such as disease treatment or management (Radbruch *et al.* 2020). Except at the end of life, palliative interventions typically are offered concurrently with disease-directed treatment. Generally, they are provided in the same doses and at the same intensity as those used for patients with non-NET cancers. Importantly, the principles underlying their use are similar across palliative populations: patient decision-making based on whether the benefits of treatments outweigh their burdens; an alignment of clinical and advance care with patient values, preferences, and goals (this includes advance care planning) (Sudore *et al.* 2017); in addition to the physical consideration of the emotional, relational, and spiritual aspects of care; the principle of ‘double effect’ (an undesired but known complication, up to and including death, of a treatment given to benefit a patient is acceptable ethically) (Potter *et al.* 2021); symptoms derived from the disease or its treatment; the use of time-limited trials; a preference to achieve multiple goals with a single intervention; and recognition that the best treatment for most symptoms is the effective treatment of the underlying disease.

For patients with NETs in particular, however, there are several special considerations around the use of palliative treatments (Singh *et al.* 2017). First, the acuity vs chronicity of the underlying NET may impact decision-making regarding palliative interventions. The assessment of the burden and risks of treatments may vary between those with aggressive disease with limited life expectancy compared with those with indolent disease expected to live many years or decades with indolent NET (Hui & Bruera 2020, Mo *et al.* 2021). For some patients with particularly aggressive NETs (life expectancy of months to a year or two), palliative care needs are very similar to those with other aggressive, life-threatening cancers. As such, many of the interventions and their intensity are similar. However, for some patients, their diagnosis of NET may be a long-term, nearly chronic challenge, with palliative interventions limited by the reality of the cost/benefit consideration. For example, opioids are typically not indicated for chronic pain due to the risks of endocrinologic complications, opioid misuse, and concerns about the efficacy of these analgesics for long-term pain. As a result of these challenges, opioids may not be indicated for pain in the setting of long-term NET (Dowell *et al.* 2016). However, for severe symptoms, opioids may become a mainstay of palliative treatment, especially in those with aggressive NET disease likely to cause the death of the patient before significant complications can accumulate. Second, given that diarrhea is a major burdensome symptom for many patients with NETs (including due to carcinoid syndrome, cholecystectomy, partial colonic resection, and/or pancreatic insufficiency), the negative side effect of opioids as analgesics for people with non-NET cancers (i.e. constipation), often becomes a *positive side effect* for people with NETs complicated by both diarrhea and pain (Stanciu & Gnanasegaram 2017). Analgesic doses of opioids may become a major component of an antidiarrheal regimen. Third, a number of symptoms seen commonly in patients with NET secondary to hormonal secretion are relatively rare in patients with other cancers, including flushing, hypertension, hypoglycemia, and symptomatic nutritional deficiencies.

### Indications for the use of SSAs in palliative care and end-of-life care

Most well-differentiated NETs express high levels of somatostatin receptors. SSAs (SSAs), including octreotide and lanreotide, bind to somatostatin receptors and can reduce hormone secretion and slow tumor growth (Modlin *et al.* 2010). In a study by Kvols *et al.* and Fisher *et al.,* the efficacy of SSAs for the treatment of carcinoid syndrome was first noted with short-acting octreotide at a dose of 150 µg subcutaneously three times per day; 88% of patients experienced an improvement in flushing and diarrhea, and 72% achieved a reduction in serotonin secretion as measured by reduction in urinary levels of its breakdown product 5-hydroxyindolacetic acid (5-HIAA) (Kvols *et al.* 1986, Fisher *et al.* 2018). Effective long-acting formulations of octreotide and lanreotide have been developed and have eliminated the need for many patients to self-administer daily injections (Rubin *et al.* 1999, Pavel *et al.* 2017, Fisher *et al.* 2018). Octreotide long-acting release (LAR) is administered as an i.m. injection every 4 weeks, and lanreotide depot is administered as a deep subcutaneous injection every four weeks. Subcutaneous short-acting octreotide can be administered to improve breakthrough symptoms of carcinoid syndrome or in situations when long-acting SSAs are not available (Pavel *et al.* 2017).

In addition to their antisecretory effects, octreotide and lanreotide can slow tumor progression. The antiproliferative effects of SSAs were demonstrated in two phase III trials. In the PROMID trial, octreotide LAR 30 mg was associated with an improvement in time to disease progression compared to placebo in patients with advanced midgut NET (Rinke *et al.* 2009). In the CLARINET trial, lanreotide depot was associated with improvement in progression-free survival compared to placebo in patients with advanced gastrointestinal and pancreatic NETs (Caplin *et al.* 2014). Due to their efficacy, ease of administration, and tolerability, SSAs are generally considered as first-line therapeutic option in well-differentiated gastroenteropancreatic NETs (GEP-NETs). The role of continuing SSAs after disease progression is debatable (Strosberg *et al.* 2017, Halfdanarson *et al.* 2020). The role of continuing SSAs after disease progression in those patients with nonfunctional NET is more controversial; generally, most physicians recommend SSAs can be discontinued in situations when treatment is no longer providing disease control or clinical benefit. For patients with functional NETs, SSAs are typically continued to minimize hormone secretion and hormone-related symptoms. Importantly, for patients receiving end-of-life care (often in hospice), continuing either long-acting formulations of SSAs or short-acting octreotide subcutaneously are appropriate strategies for minimizing hormone-related symptoms and optimizing the quality of life.

### Management of diarrhea for palliative care and end-of-life

Determining the underlying cause of diarrhea in patients with NETs is critical because diarrhea can be related to other causes, such as pancreatic insufficiency related to prior pancreatic resection or SSA therapy, effects of bowel resection, bile-acid induced diarrhea, or other gastrointestinal issues (Eads *et al.* 2020). Therefore, it is essential to exclude or treat these causes of diarrhea. In addition, some patients with carcinoid syndrome-associated diarrhea may have symptoms that become refractory to SSA over time. An option in this situation includes adding telotristat ethyl, an oral inhibitor of serotonin synthesis. In the phase III TELESTAR clinical trial, patients with carcinoid syndrome experiencing four or more bowel movements per day (BMs/day) while on SSAs were randomized to receive either telotristat ethyl or placebo. Telotristat ethyl resulted in a 42–44% mean reduction in daily bowel movements compared with placebo (Kulke *et al.* 2017). Additional strategies to improve carcinoid syndrome diarrhea have included increasing the dose or frequency of SSAs, addition of short-acting subcutaneous octreotide for breakthrough symptoms, and use of antidiarrheal therapies including loperamide, diphenoxylate-atropine, deodorized tincture of opium, or other nonspecific medications.

Nutrition and metabolism are altered in many patients with GEP-NETs. Among patients with carcinoid syndrome, the risk of malnutrition is due to reduced food intake, food intolerance, malabsorption, and diarrhea (Artale *et al.* 2020, Laing *et al.* 2020). These complications can impact patients' quality of life and functioning. Fat-soluble vitamins and niacin deficiency exist among patients with NET, particularly those on treatment with SSAs (Bouma *et al.* 2016, Lind *et al.* 2016). For patients with diarrhea, a low fiber/low residue diet with small, frequent meals is an important management component. Some food, such as amine-rich substances, can trigger carcinoid syndrome and should be avoided. The involvement of a nutritionist to assess individual needs and requirements regarding nutritional supplementation is recommended.

### Management of pain for palliative care and end of life

Pain is a common symptom in NETs, experienced by about half of the patients, and can have a debilitating effect on the quality of life (Wolin *et al.* 2017, Hallet *et al.* 2019*b*). All NET patients should continually undergo comprehensive assessment to look for reversible causes of discomfort and whenever possible patient management should be tailored based on the underlying cause (Jin *et al.* 2018). Treatment of pain in NETs is similar to other advanced cancers and has traditionally been guided by the WHO analgesic ladder (Anekar & Cascella 2023). Non-opioid analgesics such as acetaminophen (used with caution in patients with high liver burden) or ibuprofen are appropriate for mild to moderate pain (WHO 2019). Most cancer patients will require pain management therapy using a strong opioid such as morphine, oxycodone, or hydromorphone for moderate to severe pain (Chapman *et al.* 2020). The selection of opioids will depend on a patient's comorbidities as well as preferences around goals of care. Patients with dysphagia, intractable nausea, vomiting, or diarrhea may prefer a non-oral route. Transdermal fentanyl is one option; however, it is temperature-dependent and highly lipophilic, requiring adequate adipose tissue for absorption. It may not be an appropriate choice in febrile or cachectic patients at the end of life. Continuous subcutaneous administration of a parenteral opioid such as morphine or hydromorphone through a pump may be preferable for these patients (WHO 2019). Regardless of the choice of opioid or route of administration, it is essential to assess patient response after initiation of a pain regimen and adjust as patients progress through their illness.

### Management of functional neuroendocrine tumors for palliative care and end of life

Patients with metastatic pheochromocytoma/paraganglioma (mPPGL) tumors can have continuous high secretion of catecholamines and metanephrines. These hormones cause difficulty to control hypertension, especially in the setting of widespread metastatic disease. Although physicians may be less concerned about potential cardiovascular events at the end of life, hypertensive urgency can lead to discomfort for patients causing severe headaches, in particular. In addition, the high catecholamines/metanephrines can lead to severe debilitating orthostasis, diaphoresis, palpitations, flushing, and can lead to gastrointestinal ileus and/or severe constipation, which may require palliative surgical intervention (Fishbein *et al.* 2021). When discussing palliative care and end-of-life care, consideration should be given to the treatment of these symptoms for those with functional mPPGL, including potentially inhibiting catecholamine production with metyrosine or blocking effects of catecholamines with phenoxybenzamine or other alpha-adrenergic blockers.

The most common functional pancreatic neuroendocrine tumors (PNETs) are insulinomas associated with hypoglycemia with symptoms of confusion, diaphoresis, syncope and even hastened death from stroke or MI. Although rarer, other functional PNETs can have profound symptoms, such as gastrinomas (associated with Zollinger-Ellison’s syndrome (ZES) with multiple peptic ulcers, diarrhea, heartburn, weight loss); somatostatinomas (associated with diabetes mellitus, cholelithiasis, diarrhea/steatorrhea); glucagonomas (associated with weight loss, skin rash (necrolytic migratory erythema), glucose intolerance/diabetes); VIPomas (associated with profound watery diarrhea, hypokalemia, hypochlorhydria or achlorhydria, abdominal pain). The symptoms from these functional tumors can severely limit patient functional status and can be difficult for palliative care treatment and at the end of life for caregivers and patients to maintain hygiene and prevent infection. Furthermore, NETs of any kind (GI, pancreatic, lung, pheochromocytoma/paraganglioma) also can make ectopic hormones such as adrenocorticotropic hormone, leading to Cushing’s Syndrome with associated severe hypertension, abnormal weight gain, hyperglycemia, hypercoagulability, or parathyroid hormone-related peptide leading to severe hypercalcemia. All these various functional NETs diminish the quality of life and require treatment in the palliative care setting, as well as end of life, depending on the severity. For example, SSAs and other medications for profound diarrhea may be necessary even at the end of life for those with several functional PNETs. In patients with symptoms related to ZES, proton pump inhibitors (omeprazole, pantoprazole) may be needed for patient comfort. Management of severe hypoglycemia related to insulinoma at end of life, besides SSAs, glucocorticoids such as dexamethasone, boluses of IV D50W or glucagon IV or IM may be given (Gonzalez *et al.* 2015, Kok & Lee 2016). Furthermore, diazoxide is used to manage hypoglycemia by inhibiting insulin secretion; however, it can cause significant edema and it may require the use of loop diuretics (Goode *et al.* 1986, Gill *et al.* 1997, Hirshberg *et al.* 2005).

### Management of anxiety and depression related to hormonal syndromes for palliation and at the end of life

Symptoms of anxiety and depression are normal given the uncertainties of living with NETs at all stages, including at the end of life. In patients with small intenstine (SI) NET, the prevalence of depression and anxiety is 50 and 35%, respectively. These symptoms may be due to various causes, including the release of biogenic amines (5-HIAA) (Oberg 2012, Mota *et al.* 2016). In addition to the management of hormone excess with SSA (or other medications for other functional NETs), first-line antidepressants such as SSRIs appear safe in NET patients with and without carcinoid syndrome (Hemminki & Li 2001, Nobels *et al.* 2016, Isenberg-Grzeda *et al.* 2018).

La Salvia *et al.* study showed that mood disturbances, including depression and anxiety, psychoses, cognitive impairment, and sleeping alterations, are reported in NET patients, especially in patients with carcinoid symptoms, and negatively impact health-related quality of life and are associated with reduced survival rates (La Salvia *et al.* 2021). Pheochromocytomas and paragangliomas also are associated with a higher risk of anxiety and depression (Jia *et al.* 2021). The principal cause is the dysfunction of the noradrenergic system (Mineur *et al.* 2018). Consequently, early medical intervention to achieve remission of the symptoms can improve the patient's physical and mental well-being.

### Management of mesenteric fibrosis for palliation and at the end of life

Mesenteric fibrosis (MF) can affect patients with SI-NETs. Many SI-NETs either present or develop mesenteric lymph node metastases (despite often having a small primary tumor of <1cm). The lymph node metastasis can grow silently and often induce MF in the surrounding tissue of the mesentery. SI-NETs can release several growth factors and vasopeptides that can cause various symptoms such as flushing and diarrhea but can also cause tissue damage such as carcinoid heart disease and fibrosis leading to MF. MF may occur in up to 50% of SI-NETs with encasement of prominent blood vessels such as the superior mesenteric vessels (Ohrvall *et al.* 2000, Druce *et al.* 2010). MF can create contraction and tethering of bowel loops, obstruction, intussusception, and possibly ischemia. This can produce severe pain and discomfort (especially postprandial), diarrhea, ascites, malabsorption, and malnutrition, as well as ischemic-related complications. MF can significantly impact the quality of life of patients with NETs and especially at the end of life. Unfortunately, it is not well-diagnosed or recognized in many patients with SI-NET (Koumarianou *et al.* 2020).

Surgical management remains the mainstay for the treatment of MF in those with SI-NETs. Locoregional surgery can provide significant symptomatic relief. Surgery should be considered even in the presence of metastatic disease. Although surgery may lead to symptomatic relief, if done early, may result in a survival advantage (Koumarianou *et al.* 2020). It should be noted surgery may be difficult in cases involving the mesenteric vessels. When palliation and symptomatic relief is the solitary goal of care (e.g. at the end of life), attention should be paid to minimally invasive techniques such as laparoscopic surgery where possible in order to reduce the impact of the surgery on the patient with minimal recovery time (Hallet *et al.* 2021).

SSAs are known to exhibit anti-tumor activity on SI-NETs. Additionally, SSAs may reduce vasoactive peptides such as 5-HT and result in symptomatic relief and delayed onset of fibrosis (Koumarianou *et al.* 2020). SSA use may not only delay the onset of MF, but it may prevent its worsening and lead to symptomatic improvement. Therefore, SSAs are an important part of palliation for SI-NET patients with MF.

Other treatments for NETs, including telotristat ethyl, molecularly targeted agents, and peptide receptor radionuclide therapy, may delay the onset of MF. Still, their role in the acute symptomatic relief of patient symptoms at the end of life is unknown. Currently, SSA use (short-acting and long-acting) remain the mainstay for palliative symptom improvement in end-of-life treatment for patients with SI-NET.

### Management of hormone-related swelling and edema for palliation and at the end of life

Swelling and edema in patients with NETs may be related to uncontrolled hormonal secretion (VVinik *et al.* 2000). Still, other causes should be considered, including hypoalbuminemia status due to malabsorption, volume overload related to advanced carcinoid heart disease, and venous compression due to bulky retroperitoneal nodes. Ascertainment of the correct diagnosis is critical for optimal management. Diagnostic investigations include albumin/pre-albumin level, 5-HIAA, N-terminal pro-B-type natriuretic peptide, (NT-proBNP), serum serotonin, cardiac echocardiogram with a careful view of tricuspid and pulmonary valves and multiphase CT of the abdomen and pelvis to rule out venous compression. If an uncontrolled carcinoid heart disease is suspected, judicious use of diuretics is recommended as right-sided heart failure is a pre-load dependent state, and patients are at risk of hypotension (Bernheim *et al.* 2007). Cardiology consultation should be considered.

Edema and swelling-related hormonal excess may occur in the setting of progressive or increasingly functional tumors, and medical management should be maximized if tumor debulking with surgery or liver-directed therapy is not possible. Dose escalation of long-acting SSA therapy has been shown to improve symptom control and is an appropriate first-line therapy, as is the use of short-acting octreotide (Al-Efraij *et al.* 2015). Use of the telotristat which improves carcinoid syndrome-related diarrhea is also helpful in a significant reduction in other carcinoid syndrome symptoms as high serotonin may be a contributor to swelling and edema (Pavel *et al.* 2018). Judicious use of diuretics may be considered with caution to avoid hypovolemia as patients are at risk of hypotension-related vasoactive amine release. Subcutaneous interferon-alpha has been shown to palliate advanced carcinoid syndrome, including swelling. It is associated with hematologic toxicities and flu-like side effects and may be considered for refractory patients (Shah & Caplin 2005).

### Indications for palliative surgery

For many patients suffering from NETs, surgical intervention is by definition, palliative (i.e. to improve symptoms and quality of life, but not necessarily effect a cure). Although surgical eradication of the disease is possible, many patients present with stage IV disease and as such, surgical cure is not possible (Goretzki *et al.* 2018, Koea & Commonwealth Neuroendocrine Tumour Research Collaborative Surgical 2021, Niederle *et al.* 2021). Depending on the health and well-being of the patient at the time of presentation, many patients with unresectable diseases are suitable for non-curative *surgery to treat symptoms*. The issue of surgery near the end of life for patients with NETs is complex and highly selective. As with all palliative measures, the utilization of surgical intervention for improvement in symptoms must be balanced against the biological aggressiveness of disease and quality of remaining life. In a recent study, Hallet *et al* found moderate to serve symptoms of tiredness, loss of appetite, shortness of breath, nausea, and pain in NET patients during their last 6 months of life. Many of these symptoms steeply increased, especially in the last 8 weeks of life, providing for the first time some insight into what we as caregivers need to address in the terminal months of a NET patient’s life (Hallet *et al.* 2019*a*).

Although NETs are diverse in their biological aggressiveness and symptom presentations, there are a few generalizable rules for the role of palliative procedures by interventional radiology, gastroenterology, and surgery near the end of life.

- Symptomatic relief from fluid accumulation with percutaneous thoracentesis and paracentesis of pleural effusions and ascites should be utilized whenever possible.
- Surgical resection, bypass, or diversion (ostomy formation) or endoscopic stenting of intraluminal tumors causing obstruction can be considered. These interventions, however, are highly selective and should not be utilized in the face of diffuse peritoneal disease or multiple sites of obstruction.
- Biliary stenting for common bile duct obstruction can provide relief of symptoms arising from jaundice. Although commonly utilized for adenocarcinoma of the pancreas, there is limited data on pancreatic NETs (Boulay & Parepally 2014).
- Potential relief of intestinal ischemia and venous stasis of the intestine with Superior Mesenteric Vein (SMV) stenting has been reported, yet it is highly selective (Daskalakis *et al.* 2017). In a small series of 20 patients with SMV occlusion, the authors utilized endovascular SMV stents in 12 patients with a third reporting resolution of their symptoms.
- In the case of obstructive uropathy causing hydronephrosis from MF, percutaneous J-stents can provide excellent palliation (Daskalakis *et al.* 2017).

## End-of-life consideration for patients with NETs

### What distinguishes NETs from other cancers at end-of-life?

In the last past few decades, the annual incidence of NETs has continued to rise in the United States and worldwide. Dasari *et al.* reported that the incidence of NETs had risen from 1 in 100,000 persons per year in the 1970s to 6.98 in 100,000 persons per year in 2012 (Dasari *et al.* 2017). More than 12,000 new cases are diagnosed each year and approximately 125,000 people are living with these tumors. NETs are a relatively rare disease, comprising ~2% of all malignancies, making it a rare disease in the United States and other parts of the world (Oronsky *et al.* 2017). NETs are unique because they can produce and secrete hormones (functional NETs); however, patients can also have nonfunctional NETs. Small intestinal neuroendocrine cancers (SI-NETs) and bronchial NETs are often associated with excess serotonin secretion measured by elevation in urinary levels of its breakdown product 5-HIAA, causing profound diarrhea and organ fibrosis. Functional PNETs also occur in about 10–15% of cases, leading to various syndromes depending on the hormone secretion (Halfdanarson *et al.* 2020). Pheochromocytoma and paraganglioma can secrete catecholamines and metanephrines, leading to profound hypertension, hyperglycemia, and other complications.

Patients with NETs spend years searching for the correct diagnosis, and then they may spend even more time finding a specialist who understands the tumor and the symptoms it can produce. While the current NANETS and NCCN® (National Comprehensive Cancer Network®) guidelines provide guidance on the management of symptoms that these tumors can produce, there is no guidance about how to manage symptoms related to hormone excess as a patient enters hospice end-of-life care (Chan *et al.* 2018, Halfdanarson *et al.* 2020, Hope *et al.* 2020, Fishbein *et al.* 2021). Moreover, a NET patient may have difficulties finding a palliative or hospice medical team that understands their unique symptoms and how to manage them. Patients with NETs may encounter discouragement at the most vulnerable moment in their lives due to the paucity of knowledge on managing NET-related symptoms at end of life.

## Special considerations around payment and coverage at end of life (including medications) in patients with NETs

In 2021, on average, a hospice is paid $199.25 per patient per day for the first 60 days of the hospice benefit. After this, it is reduced to $157.49, on average, per patient day (CMS 2021). In 2018, a hospice patient's average length of stay was 77.9 days (CMS). The medical interventions we have mentioned above cost anywhere from $250 per day to $1000 per day. It is clear that a hospice care organization is not funded to provide the interventions that may be needed for patients with NETs to receive the best care possible toward the end of life.

We encourage:

- Drug manufacturers to consider accommodations for patients needing these drugs while in hospice care.
- Hospice providers to reach out to drug manufacturers for patient accommodation as a patient enters hospice.
- Physicians should be encouraged to consider the benefit of those in the context of their burdens and fully and realistically explain those benefits and potential cost to their patients.

When considering surgical interventions for hospice-eligible patients, the above information about payment structure should be considered. In addition to this, hospices with high rates of revocation followed by readmission are at risk of increased scrutiny making this option unattractive. As mentioned earlier, physicians should be encouraged to consider the benefit of surgical interventions in the context of their burdens and fully and realistically explain those burdens in relation to benefits to their patients.

## Summary

Patients with neuroendocrine neoplasms have unique disease-related symptoms that impact palliative care needs during routine disease management and at the end of life. There are special considerations around the use of palliative treatments when caring for patients with advanced neuroendocrine neoplasms that should be taken into account: disease biology and prognosis, as well as symptoms that may be related to location and burden of disease and/or hormone secretion. SSAs can palliate symptoms of hormone excess, such as flushing and diarrhea, that impact quality of life; however, there remain challenges related to cost and access to SSA therapy for patients receiving hospice care. Patients with pheochromocytoma/paraganglioma and functional pancreatic NETs also may have profound symptoms that require specific palliation during the course of their disease and at the end of life. Other issues that are unique to patients with advanced NETs include MF and bowel or biliary tract obstruction that may require intervention. Awareness of these issues and other important medical and cost issues covered in this white paper will allow well-informed discussion and multidisciplinary care to maximize the patient quality of life during routine care and at the end of life.

## Declaration of interest

Hagen Kennecke received <$5000 for participation in advisory boards for Terseara, Novartis and Natera; Janice Pasieka held seats on the medical advisory boards for Ipsen and Novartis; Simron Singh recieved honorarium from Ipsen and institutional grant from Novartis/AAA; Lauren Fishbein served as Lantheus/Azedra consultant; Josh Mailman received <$5000 sponsorship for advisory roles from Crinetics, Rayzbio, Camurus, Ipsen, Curium, Tersera. Other authors had no conflict of interest.

## Funding

This work did not receive any specific grant from any funding agency in the public, commercial or not-for-profit sector.
